# Bone health in advanced prostate cancer: pathophysiology and management strategies

**DOI:** 10.1093/oncolo/oyag321

**Published:** 2026-08-25

**Authors:** Rana R McKay, Heather Hofflich, Gina Woods, Richard Andres, Azeez Farooki

**Affiliations:** Department of Medicine and Urology, University of California San Diego, San Diego, CA 92093, United States; Department of Medicine, University of California San Diego, San Diego, CA 92093, United States; Department of Medicine, University of California San Diego, San Diego, CA 92093, United States; Bayer HealthCare Pharmaceuticals, Whippany, NJ 07981, United States; Department of Medicine and Endocrinology, David H. Koch Center for Cancer Care, Memorial Sloan Kettering Cancer Center, New York, NY 10021, United States

**Keywords:** prostate cancer, bone health, bone health agents, health outcomes, quality of life

## Abstract

Bone loss and bone metastases can increase morbidity and impair quality of life for patients with prostate cancer. Use of hormonal agents can compromise bone integrity from treatment initiation, and the emergence of bone metastases further increases the risk of symptomatic skeletal-related events. Management of bone health should therefore be pivotal across the prostate cancer continuum. Current guidelines recommend that all men starting or receiving androgen-deprivation therapy for prostate cancer be assessed regularly for bone loss using predictive tools such as dual-energy X-ray absorptiometry and Trabecular Bone Score. Bone health agents (BHAs) are recommended for all men with castration-resistant prostate cancer with skeletal metastases to prevent symptomatic skeletal-related events and for high-risk men receiving androgen-deprivation therapy to prevent treatment-related bone loss. Options include bisphosphonates such as zoledronic acid and alendronate and the receptor activator of nuclear factor kappa-B ligand inhibitor denosumab. Patients should be monitored for adverse effects of BHAs, such as osteonecrosis of the jaw and hypocalcemia. Alongside BHAs, there are treatment options for metastatic castration-resistant prostate cancer associated with beneficial bone-related effects, notably abiraterone acetate, enzalutamide, and radium-223. In this narrative review, we focus on how bone health management can be integrated into standard clinical practice in men with advanced prostate cancer. We also present a test-and-treatment algorithm for bone health management in men with metastatic prostate cancer. Improving treatment-related bone loss and proactively managing bone metastases should be standard practice in all men with prostate cancer to improve health outcomes and quality of life.

## Graphical abstract

**Graphical Abstract:**
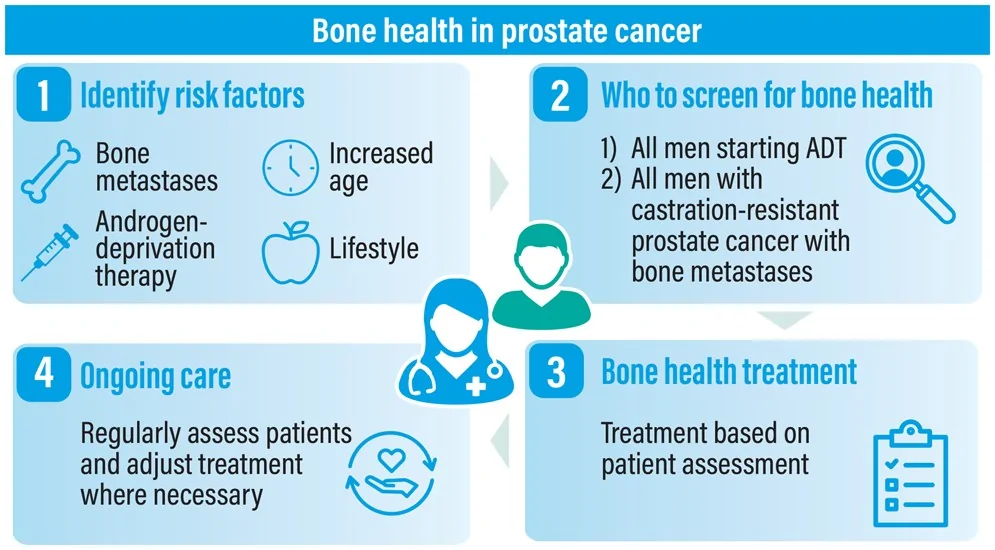

Implications for Practice Bone loss and bone metastases increase morbidity and impair quality of life for patients with prostate cancer. Bone health agents are recommended for many such patients but are underused. Some treatments for metastatic castration-resistant prostate cancer, notably abiraterone acetate, enzalutamide, and radium-223, have beneficial bone-related effects. This review discusses the pathophysiology, treatment options, and risk assessments in relation to bone health in men with advanced prostate cancer and proposes a practical algorithm for bone health management in this setting.

## Introduction

Bone health is negatively impacted across the prostate cancer continuum. In localized disease, hormonal agents reduce testosterone levels and accelerate bone loss, thereby increasing the risk of fragility fractures.^1^ In advanced disease, bone metastases lead to further complications.^2^ Approximately 10% of patients present with bone metastases at initial diagnosis^3^ and approximately 85% at diagnosis of metastatic castration-resistant prostate cancer (mCRPC),^4^ often leading to symptomatic skeletal-related events (SSEs), including pathologic fracture, spinal cord compression, and requiring external beam radiation or surgery to manage bone pain.^2^ SSEs are associated with reduced quality of life and increased pain, morbidity, and mortality.^5^ In men with mCRPC, fracture risk and declining bone mineral density are further accelerated with increasing age and treatment exposure, including steroids and hormonal therapy.^6^ The recommended treatments for mCRPC are abiraterone acetate plus prednisone, sipuleucel-T, docetaxel, cabazitaxel, enzalutamide, radium-223, and lutetium-PSMA-617,^7^ based on proven survival benefits.^8–14^ While some treatments that inhibit androgen production may adversely affect bone health, abiraterone acetate plus prednisone, enzalutamide, and radium-223 reduce SSE risk.^12^^,^^15^^,^^16^

Bone health agents (BHAs) are recommended in prostate cancer management guidelines for men at high risk of fracture receiving androgen-deprivation therapy (ADT) to prevent treatment-related bone loss and in men with mCRPC with skeletal metastases to prevent SSEs.^7^^,^^17^ However, BHAs are under-used in clinical practice. In a real-world US study (*n *= 1 292; 2007-2015), one-third of men with mCRPC and bone metastases were not receiving BHAs.^18^ A more recent US study (*n *= 14 112; 2013-2024) reported that use of BHA in men with mCRPC decreased from 56% in 2013 to 46% in 2024.^19^ Similarly, in a study of men with mCRPC in the Netherlands (*n *= 1 923; 2010-2015), only 52% received early initiation of BHAs despite the lower incidence of SSEs and longer SSE-free survival observed with this strategy.^20^ In another European real-world study, oncologists were more likely to prescribe BHAs than urologists (78% versus 60%), suggesting that physician education may be required for optimal patient care.^21^ Barriers to use of BHAs operate at many levels. At the patient level, adherence can be limited by the burden of repeated injections or infusions and additional clinic visits layered onto an already demanding oncologic regimen, the need to complete dental work before initiation, and concern over adverse events. These include osteonecrosis of the jaw (ONJ), hypocalcemia, and, with intravenous bisphosphonates, acute-phase reactions.^22–25^ Because bone loss is itself asymptomatic, patients may be reluctant to accept these risks for a benefit they cannot perceive. At the clinician level, underuse may reflect limited awareness of guidelines, ambiguity over which physician is responsible for initiating and monitoring bone-directed therapy, uncertainty over correct agent and dose, and insufficient clinic time to address bone health. At the system level, barriers include the need to obtain dental clearance before treatment, lack of support systems and local guidelines, and inadequate reimbursement or insurance coverage.^26–29^

Given the systematic underuse of BHAs, this narrative review synthesizes evidence-based recommendations for bone health management in men with advanced prostate cancer into a practical guide for clinicians. The review also explores bone physiology and why BHAs should be used as standard of care for prevention of bone loss, fragility fractures, and SSEs.

## Bone physiology

The skeleton undergoes continuous remodeling, a dynamic process of bone formation by osteoblasts and bone resorption by osteoclasts, which helps maintain bone strength and mineral homeostasis.^1^^,^^30^ Osteoporosis is a systemic skeletal disorder characterized by low bone mass and microarchitectural deterioration of bone tissue.^31^ This can be triggered by a variety of factors, including hormonal changes, aging, or disease.^30^^,^^31^

**Figure 1.**
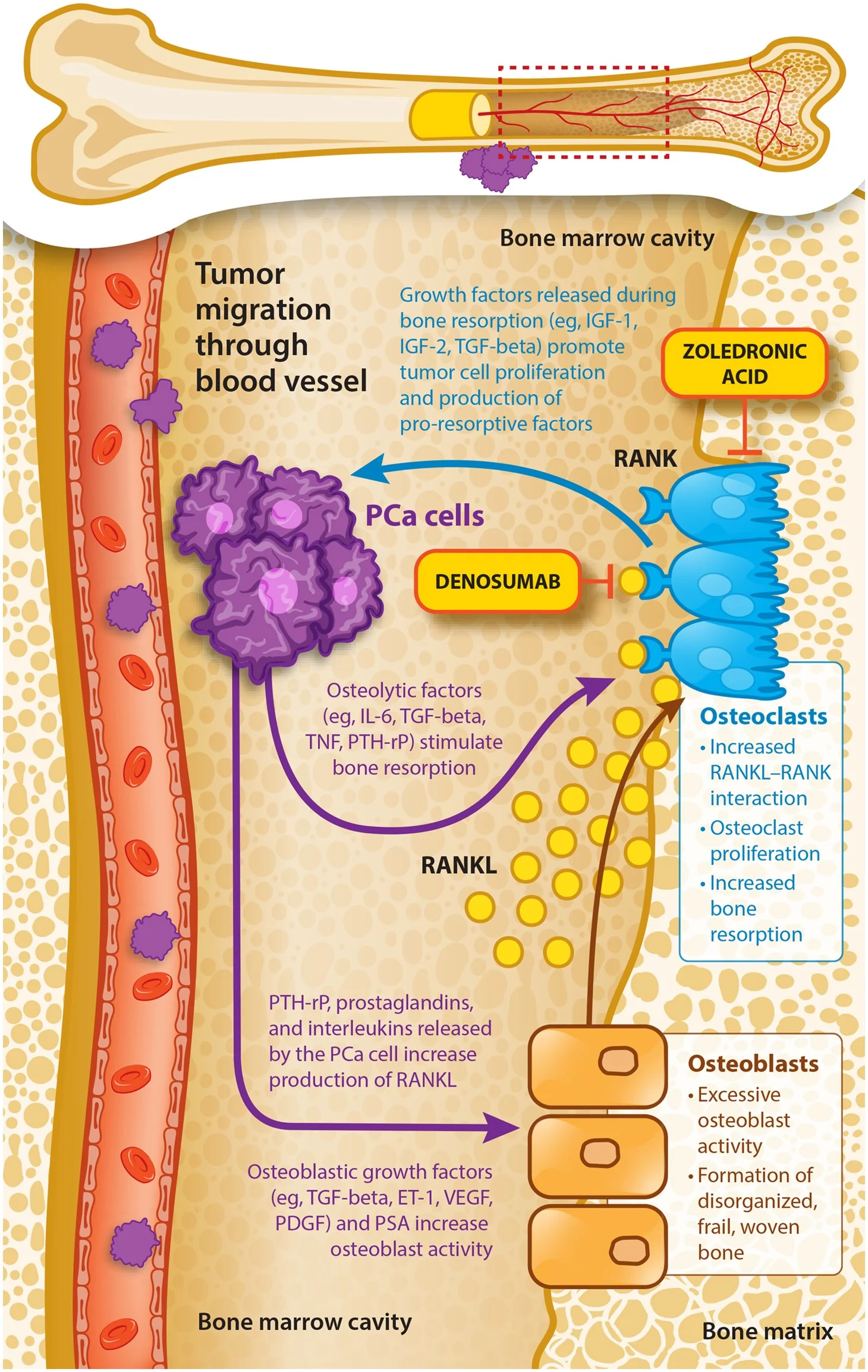
Bone metastases disturb the relationship between osteoblastic bone formation and osteoclastic bone resorption, resulting in a cycle of bone destruction and tumor growth. Bone health agents such as zoledronic acid and denosumab can be used to preserve bone health by inhibiting osteoclast activity.^30^^,^^32–36^ ET-1, endothelin-1; IGF, insulin-like growth factor; IL, interleukin; PCa, prostate cancer; PDGF, platelet-derived growth factor; PSA, prostate-specific antigen; PTH-rP, parathyroid hormone-related protein; RANK, receptor activator of nuclear factor kappa-B; RANKL, receptor activator of nuclear factor kappa-B ligand; TGF, transforming growth factor; TNF, tumor necrosis factor; VEGF, vascular endothelial growth factor.

Bone metastases disturb the relationship between osteoblastic bone formation and osteoclastic bone resorption, resulting in a cycle of bone destruction and tumor growth (Figure 1).^1^^,^^32^ The receptor activator of nuclear factor kappa-B ligand (RANKL), expressed on osteoblasts and stromal cells, is a critical cytokine in the bone remodeling process.^30^ RANKL binds to RANK receptors on the surface of osteoclast progenitors, triggering a signaling cascade that results in osteoclast maturation and bone resorption.^30^^,^^33^ Parathyroid hormone-related protein and growth factors secreted by tumor cells stimulate osteoblast activity, resulting in an increased expression of RANKL, leading to osteoclast proliferation and increased bone resorption.^33^^,^^34^ The release of bone morphogenetic proteins and growth factors from the bone matrix after bone resorption further drives the cycle of tumor metastasis and osteolysis.^33^^,^^34^

Bone metastases in prostate cancer are usually osteoblastic, partly due to promotion of osteoblastic proliferation by prostate-specific antigen and endothelin-1, both of which are elevated in men with prostate cancer (Figure 1).^35–37^ N bone in osteoblastic lesions presents as irregular, woven bone that is prone to fracture.^1^^,^^36^ Systemic markers of bone resorption, such as pyridinoline cross-linked carboxyterminal telopeptide and C- and N-terminal telopeptides of type I collagen, are higher in patients with bone metastasis than those without.^30^^,^^38^^,^^39^ Additionally, osteoclast activation is important; bone resorption precedes bone formation in the development of osteoblastic metastases from primary prostate cancer.^30^^,^^40^

## Treatment-related bone loss and skeletal-related events (SREs)

Treatment factors (eg, agent and duration of use) and patient factors (eg, age, body habitus, lifestyle factors, and fitness) can affect bone integrity.^41^ In a real-world study in the USAADT monotherapy and ADT combinations were used in > 50% and 36% of patients with metastatic hormone-sensitive prostate cancer (mHSPC), respectively.^42^ADT substantially reduces androgens and estrogens, both essential in regulating bone integrity.^1^ Within 6 months of ADT initiation, men have a 5 to 10-fold greater loss of bone density compared with either healthy men or men with prostate cancer not receiving ADT.^43^ In men with non-metastatic prostate cancer (*n *= 89), 12 weeks of ADT therapy resulted in elevated markers of bone loss as early as 3 months post-initiation.^44^

Androgen receptor pathway inhibitors combined with ADT (eg, abiraterone acetate) can further increase the risk of bone loss and fractures compared with ADT alone, partly due to further reduction in androgens and estrogens but also through concomitant low-dose steroids.^45^ In a recent randomized trial, the addition of abiraterone acetate plus prednisone to ADT did not increase bone loss significantly, but a numerically higher rate of osteoporosis was observed at 2 years.^45^ Long-term use of glucocorticoids is the most common cause of secondary osteoporosis due to increased bone resorption, decreased bone formation, and interruption of bone regulatory pathways.^46^ While potent androgen receptor suppression is crucial for prostate cancer treatment, it is essential to be aware of potential treatment-related bone loss and take proactive measures to manage bone health.

In addition to increased bone loss, prostate cancer treatment can increase fracture risk. In large registry studies in the USA and Canada, ADT increased fracture risk by over 30%.^47^^,^^48^

## Bone health management strategies

Initial approaches for bone health management in men with prostate cancer may include education regarding lifestyle modifications and nutritional supplements. Evidence for weight-bearing exercise directly improving bone mineral density in men with prostate cancer is sparse, and the effect is inconsistent.^49–51^ However, the LIFTMOR-M semi-randomized controlled trial demonstrated that high-intensity resistance and impact training improves bone mineral density in middle-aged and older men with osteopenia and osteoporosis.^52^ Smoking, excessive alcohol intake, and low calcium intake are associated with lower bone mineral density in men with prostate cancer.^53^^,^^54^ Individualized lifestyle modifications, including smoking cessation, moderating alcohol consumption, ensuring sufficient calcium and vitamin D intake, regular resistance or strength training, and using strategies to mitigate falls, are therefore important for improving bone health.^1^^,^^33^^,^^46^ Increasing dietary protein intake (1.0-1.2 g/kg/day for muscle preservation in aging and 1.2-1.5 g/kg/day for those who are elderly with acute or chronic diseases) may help to counteract ADT-related skeletal muscle loss,^55^ although data for prostate cancer specifically are lacking.

Bisphosphonates are chemical derivatives of inorganic pyrophosphate, which inhibits calcification by binding to hydroxyapatite crystals.^56^ Bisphosphonates have a high affinity for bone mineral and are preferentially incorporated into sites of osteoclast-mediated bone resorption and active bone remodeling.^56^^,^^57^ Randomized trials have shown bisphosphonates to prevent ADT-associated bone loss.^58–62^ Additionally, the bisphosphonate zoledronic acid reduces SREs in men with mCRPC and bone metastases (Table 1).

**Table 1.** Overview of pharmacologic bone health management strategies for men with advanced prostate cancer.

**Zoledronic acid**	
**Prostate cancer stage**	**Evidence**
**Castration-sensitive with no bone metastases**	Increases bone mineral density Bone mineral density of the femoral neck, total hip, and lumbar spine increased over the 1-year study period by 3.6%, 3.8%, and 6.7%, respectively, with zoledronic acid versus placebo (*P* < .0004, *P* < .0001, and *P* < .0001, respectively; *n *= 120)^61^ Bone mineral density of the lumbar spine increased by 5.6% with zoledronic acid and decreased by 2.2% with placebo over 1 year (*P* < .001; *n *= 106)^62^ Does not prevent bone metastasis ZEUS study: Kaplan–Meier estimated proportion of bone metastases after a median follow-up of 4.8 years was 14.7% in the zoledronic acid group versus 13.2% in the control group (*P* = .65; *n *= 1393)^63^ Does not add survival benefit when added to standard therapy STAMPEDE study: 5-year survival in the zoledronic acid group was 57% versus 55% with ADT alone (HR, 0.94; 95% CI, 0.79-1.11; *P* = .45; *n *= 2962)^64^ RADAR study: Adjusted cumulative incidence of prostate cancer-specific mortality was 11.2% with zoledronic acid versus 11.7% without in a 10-year analysis (absolute difference of −0.5% [95% CI, −3.8 to 2.9]; adjusted *P* = .78; *n *= 1071)^65^
**Castration-sensitive with bone metastases**	Does not delay time to first SRE Median time to first SRE was 31.9 months in the zoledronic acid group versus 29.8 months in the placebo group (HR, 0.97; 95% CI, 0-1.17; *P* = .39; *n *= 645)^66^ Does not add survival benefit when added to standard therapy Overall survival was similar between the zoledronic acid group and the placebo group (HR, 0.88; 95% CI, 0.70-1.12; *P* = .29; *n *= 645)^66^
**Castration-resistant with bone metastases**	Decreases rate of SREs A greater proportion of patients in the placebo group had SREs versus those in the zoledronic acid 4 mg group (44.2% versus 33.2%; difference = −11.0%; 95% CI, −20.3 to −1.8; *P* = .021; *n *= 422)^67^ TRAPEZE study: Zoledronic acid showed a significant SRE-free interval when combined with docetaxel plus prednisolone compared with docetaxel plus prednisolone alone (HR, 0.76; 95% CI, 0.63-0.93; *P* = .008; *n *= 757)^68^ Does not add survival benefit when added to standard therapy TRAPEZE study: Zoledronic acid showed no survival benefit when combined with docetaxel plus prednisolone compared with docetaxel plus prednisolone alone (*P* = .91; *n *= 757)^68^
**Denosumab**	
**Prostate cancer stage**	**Evidence**
**Castration-sensitive with no bone metastases**	Increases bone mineral density HALT study: Bone mineral density of the lumbar spine increased by 5.6% in the denosumab group versus a loss of 1.0% in the placebo group (*P* < .001; *n *= 1468); improvements were seen as early as 1 month and sustained through 36 months^69^ Decreases the risk of vertebral fractures HALT study: Incidence of new vertebral fractures was 1.5% in the denosumab group versus 3.9% in the placebo group (relative risk, 0.38; 95% CI, 0.19-0.78; *P* = .006; *n *= 1468)^69^
**Castration-resistant with no bone metastases**	Improves bone metastasis-free survival Bone metastasis-free survival was increased by a median of 4.2 months with denosumab versus placebo (HR, 0.85; 95% CI, 0.73-0.98; *P* = .028; *n *= 1432)^70^ Time to first bone metastases was delayed with denosumab versus placebo (HR, 0.84; 95% CI, 0.71-0.98; *P* = .032; *n *= 1432)^70^ Does not add survival benefit when added to standard therapy Overall survival was similar between the zoledronic acid group and the placebo group (HR, 1.01; 95% CI, 0.85-1.20; *P* = .91; *n *= 1432)^70^
**Castration-sensitive with bone metastases**	No data available
**Table 1.** (continued)	
**Denosumab**	
**Prostate cancer stage**	**Evidence**
**Castration-resistant with bone metastases**	Increases time to first SRE versus zoledronic acid Median time to first SRE was 20.7 months with denosumab compared with 17.1 months with zoledronic acid (HR, 0.82; 95% CI, 0.71-0.95; *P* = .0002 for non-inferiority; *P* = .008 for superiority; *n *= 1901)^71^ Denosumab significantly reduced the risk of developing a first SSE (HR, 0.78; 95% CI, 0.66-0.93; *P* = .005) and first and subsequent SSE (rate ratio, 0.78; 95% CI, 0.65-0.92; *P* = .004) compared with zoledronic acid (*n *= 1901)^72^

Abbreviations: ADT, androgen-deprivation therapy; CI, confidence interval; HALT, Denosumab Hormone Ablation Bone Loss; HR, hazard ratio; RADAR, Randomized Androgen Deprivation and Radiotherapy; SRE, skeletal-related event; SSE, symptomatic skeletal-related event; STAMPEDE, Systemic Therapy in Advancing or Metastatic Prostate Cancer Evaluation of Drug Efficacy; TRAPEZE, A randomized phase III trial of docetaxel plus prednisolone versus docetaxel with prednisolone plus either zoledronic acid, strontium-89, or both agents combined; ZEUS, Zometa European Study.

Zoledronic acid is a nitrogen-containing bisphosphonate that promotes osteoclast apoptosis by inhibiting farnesyl pyrophosphate, a key regulatory enzyme in osteoclast function (Figure 1).^56^ Bisphosphonates limit osteoclast-mediated bone loss and are generally well tolerated. However, potential adverse effects may limit their use in some patients, including gastrointestinal upset with oral bisphosphonates, an acute-phase reaction consisting of fever, myalgia, and arthralgias after the first dose of intravenous bisphosphonate, hypocalcemia, ocular inflammation, acute renal insufficiency, ONJ, atypical femur fracture, and atrial fibrillation.^22–25^^,^^56^^,^^57^

Denosumab is a fully human monoclonal antibody with high affinity and specificity for RANKL.^34^^,^^69^^,^^73^ Osteoblastic proliferation in prostate cancer bone metastases increases the expression of RANKL and subsequently increases osteoclast formation and activity.^34^ Denosumab inhibits osteoclast activity and reduces bone resorption by blocking RANK and RANKL binding (Figure 1),^34^^,^^73^ and can improve bone health in men with prostate cancer (Table 1). In the phase III HALT trial of men receiving ADT for non-metastatic prostate cancer, denosumab was associated with increased bone mineral density at all measured sites and a reduction in the incidence of new vertebral fractures versus placebo.^69^ Potential adverse effects—including hypocalcemia, increased risk of infection, atypical femur fracture, and ONJ^74–76^ may limit the use of denosumab in some patients. Discontinuation of denosumab leads to a rapid rebound in bone turnover, usually causing loss of accrued bone mineral density and rarely causing an increase in the risk of multiple vertebral fractures.^75^^,^^77^

## Bone-targeted treatments for prostate cancer

### Skeletal-related events

SREs were evaluated in clinical trials of abiraterone acetate, enzalutamide, and radium-223.^11^^,^^12^^,^^15^ In the phase III COU-AA-301 study, 1195 men with mCRPC were randomized to receive prednisone with either abiraterone acetate or placebo.^78^ Compared with prednisone alone, the abiraterone acetate combination was associated with a longer median time to first SRE (25.0 months [95% confidence interval [CI], 25.0-not estimable] versus 20.3 months [16.9-not estimable]; *P* = .0001), palliation of pain intensity in more patients (45.0% versus 28.8%; *P* = .0005), and shorter median time to palliation (5.6 months [95% CI, 3.7-9.2] versus 13.7 months [5.4-not estimable]; *P* = .0018).^15^ Such effects are likely due to the propensity of abiraterone acetate plus prednisone to decrease bone resorption preferentially at the level of the metastatic site.^79^

In the phase III PREVAIL study, 1717 men with mCRPC were randomized to receive enzalutamide or placebo.^11^ Enzalutamide reduced the risk of a first SRE (hazard ratio [HR], 0.72; 95% CI, 0.61-0.84; *P* < .0001) and increased median time to pain progression (HR, 0.62; 95% CI, 0.53-0.74; *P* < .0001).^11^^,^^16^ The effect of enzalutamide on bone health may be partly due to interactions with androgen receptors on osteoblasts and the RANKL pathway.^80^

While these novel hormonal agents are associated with lower risk for pathologic fracture, they may increase the risk of non-pathologic fracture and falls.^81^^,^^82^ In the COU-AA-301 study, rates of non-pathologic fracture were 5.9% with abiraterone acetate plus prednisone versus 2.3% with prednisone alone.^83^ In a post hoc meta-analysis of enzalutamide phase III studies, fall rates (9.1% versus 3.6%) and fracture rates (10.2% versus 4.4%) were higher with enzalutamide versus placebo.^84^ This highlights the need to regularly assess bone health.

Radium-223 is a bone-seeking calcium mimetic, selectively binding to areas of increased bone turnover and emitting high-energy alpha particle radiation, particularly within the microenvironment of osteoblastic or sclerotic metastases.^12^ In the phase III ALSYMPCA study, 921 men with mCRPC with bone metastases were randomized to receive radium-223 or placebo.^12^ Time to first SSE was significantly prolonged with radium-223 versus placebo (median, 15.6 months versus 9.8 months; HR, 0.66; 95% CI, 0.52-0.83; *P* < .001).^12^ A systematic literature review of 48 studies of radium-223 highlighted low rates of fracture with the treatment and guideline-recommended BHAs.^85^

The ERA-223 study aimed to assess radium-223 combined with abiraterone acetate plus prednisone; however, the study was unblinded prematurely after reports of a significant increase in fracture rates.^86^ Subsequently, a protocol amendment mandating BHA use was introduced to another study of radium-223, PEACE-III (enzalutamide alone or in combination with radium-223).^86^ After continuous administration of a BHA started ≥ 6 weeks before initiation of radium-223, the 1-year fracture rate was reduced from 15.6% to 2.6% in patients receiving enzalutamide monotherapy.^86^

SRE rates are an important measure of health-related quality of life in clinical trials. SRE-based outcomes have evolved from single-event measurements to composite measurements incorporating survival (eg, SRE-free survival).^87^ While overall survival remains the primary outcome in advanced cancer trials, the US Food and Drug Administration (FDA) recommends including outcomes that reflect symptomatic improvement.^88^

### Survival

In the COU-AA-301 study, addition of abiraterone acetate increased overall survival after 12.8 months versus prednisone alone (14.8 versus 10.9 months; HR, 0.65; 95% CI, 0.54-0.77; *P* < .001).^78^ In PREVAIL, enzalutamide led to increased radiographic progression-free survival (rPFS) after 12 months versus placebo (65% versus 14%; HR, 0.19; 95% CI, 0.15-0.23; *P* < .001).^11^ In the ALSYMPCA study, radium-223 was associated with improved overall survival versus placebo (median, 14.9 months versus 11.3 months; HR, 0.70; 95% CI, 0.58-0.83; *P* < .001).^12^ Final results from PEACE-III showed a statistically significant overall survival benefit with the radium-enzalutamide combination (HR, 0.76; 95% CI, 0.60-0.96; *P* = .0096), with a significant improvement in rPFS (HR, 0.71; 95% CI, 0.57-0.89; no *P* value because rPFS at the final analysis was exploratory).^89^ In an additional analysis, BHA use vs no BHA use in all patients led to a 14-month improvement in rPFS (HR, 0.60; 95% CI, 0.39-0.93) and a 17-month improvement in overall survival (HR, 0.56; 95% CI, 0.37-0.86).^90^ These data suggest that BHAs should be given routinely with radium-223 and other prostate cancer treatments to preserve bone health with improvement in treatment benefit.

#### Bone loss

In two placebo-controlled studies of zoledronic acid in patients without bone metastases, bone mineral density was significantly increased at the lumbar spine, femoral neck, and total hip after 1 year of treatment (*P* < .001).^61^^,^^62^ Significantly (*P* < .001) increased bone mineral density of the lumbar spine versus placebo was also reported in the HALT study of denosumab.^69^

## Assessing bone health and dosing of BHAs in patients with prostate cancer

All men starting or receiving ADT for prostate cancer, regardless of disease stage, should be regularly assessed for treatment-related bone loss to identify those who may benefit from BHAs.^17^ The Fracture Risk Assessment Tool (FRAX) is a computer-based algorithm that calculates the 10-year probability of major osteoporotic fracture and hip fracture.^91^ FRAX is the most used fracture risk prediction tool worldwide and has demonstrated accuracy in men with prostate cancer; importantly, no effect modification was observed with prostate cancer status or current ADT.^91^ Other evaluations of bone health include dual-energy X-ray absorptiometry screening and Trabecular Bone Score (TBS). TBS is an index calculated by textural analysis of pixel density in lumbar spine images, allowing assessment of bone quality and fracture risk independent of bone mineral density.^92^ Hounsfield Unit values of trabecular bone in axial computed tomography images of vertebral bodies derived from staging computed tomography scans can also provide information to identify men with prostate cancer who may require BHAs. These images are able to exclude regions with obvious spinal degenerative changes or aortic calcification.^93^

Baseline 25-hydroxy vitamin D should also be assessed before initiating therapy, with supplements prescribed if necessary.^17^

Another important topic is the selection and dosing of BHAs. The National Comprehensive Cancer Network 2025 guidelines recommend antiresorptive medications such as denosumab 60 mg subcutaneously every 6 months, or a bisphosphonate such as zoledronic acid 5 mg intravenously once yearly, or alendronate 70 mg orally once weekly.^17^ To prevent or delay SREs in men with mCRPC and bone metastases, 120 mg subcutaneous denosumab every 4 weeks or intravenous zoledronic acid 4 mg every 12 weeks is recommended^17^; however, optimal dosing duration in this setting remains unclear. In open-label trials, 4-week therapy of zoledronic acid or denosumab was non-inferior to 12-week therapy over 1 and 2 years, respectively.^94^^,^^95^ BHAs are not recommended for SRE prevention in mHSPC given the absence of clinical benefit and negative phase III data.^17^^,^^66^

In treated or non-active bone metastases, the decision to stop or de-escalate BHA therapy depends on the type of BHA being used and fracture risk. For patients receiving ADT treatment at high risk of fracture, BHAs should be continued until they are no longer at high risk, as determined by the ADT completion, FRAX assessment, or other risk assessments. For patients receiving zoledronic acid, treatment should be discontinued upon confirmation of low-risk status. Patients receiving denosumab who are no longer at high risk should transition to a bisphosphonate upon discontinuation to mitigate the risk of bone mineral density loss and vertebral fractures.^17^^,^^96^ There is a lack of data and guidelines for therapy de-escalation; further research is needed to support clinical practice.

## Monitoring and managing adverse effects associated with BHAs

Potential adverse effects of BHAs should be monitored throughout treatment to ensure timely, appropriate management if they occur. As bisphosphonates and denosumab are potent inhibitors of bone resorption, they are both associated with the development of ONJ,^22–25^^,^^76^ a rare but serious side effect resulting in exposed bone or bone that can be probed through an intraoral or extraoral fistula in the maxillofacial region, persisting for > 8 weeks.^97^ Among those receiving a bisphosphonate or denosumab, the risk of ONJ increases over time.^97–99^ Although no strategies eliminate the risk of ONJ, preventative steps such as smoking cessation, diabetes management optimization, maintaining good oral hygiene, avoiding invasive dental procedures where possible, and dental evaluation before and during treatment are recommended.^97^^,^^100^ In the event of ONJ, BHAs should be discontinued. In patients who develop ONJ while receiving denosumab, treatment of ONJ should be carefully balanced with the risk of rebound bone loss and vertebral fracture associated with denosumab discontinuation.^17^ ONJ can be treated with conservative measures, but for those who do not improve, surgical debridement or resection of necrotic bone may be necessary.^100^ A multidisciplinary approach involving oncologists, dentists, and oral surgeons is recommended.

Inhibition of bone resorption by bisphosphonates and denosumab can also induce hypocalcemia^22^ due to reduced calcium efflux from bone into the circulation.^101^ In a US real-world study from the FDA Adverse Event Reporting System database, denosumab showed a safety signal for hypocalcemia, particularly in elderly males and patients with cancer.^102^ In a retrospective USA-based cohort study, denosumab was associated with a higher incidence of severe hypocalcemia (41% versus 2%) and very severe hypocalcemia (11% versus 0.4%) compared with oral bisphosphonates in female dialysis-dependent patients aged ≥ 65 years.^103^ Thus, in January 2024, the FDA included a boxed warning for denosumab communicating the increased risk of hypocalcemia in patients with advanced chronic kidney disease (CKD; estimated glomerular filtration rate < 30 mL/min/1.73 m^2^),^76^ particularly in the 2-10 weeks after injection.^104^^,^^105^ A case series of severe hypocalcemia due to denosumab in patients with mCRPC found that a large burden of osteoblastic disease, vitamin D deficiency, and possibly severe calcium malabsorption were risk factors.^106^

To minimize hypocalcemia risk in patients receiving denosumab with CKD, the FDA recommends evaluating the presence of CKD–mineral bone disorder and assessing bone turnover status.^76^^,^^105^ Consultation and coordinating care with an endocrinologist or nephrologist may also be helpful. In the general prostate cancer population without CKD, steps to minimize hypocalcemia risk may include checking parathyroid hormone levels for evidence of CKD–mineral bone disorder and adequate supplementation with calcium and vitamin D.

**Figure 2.**
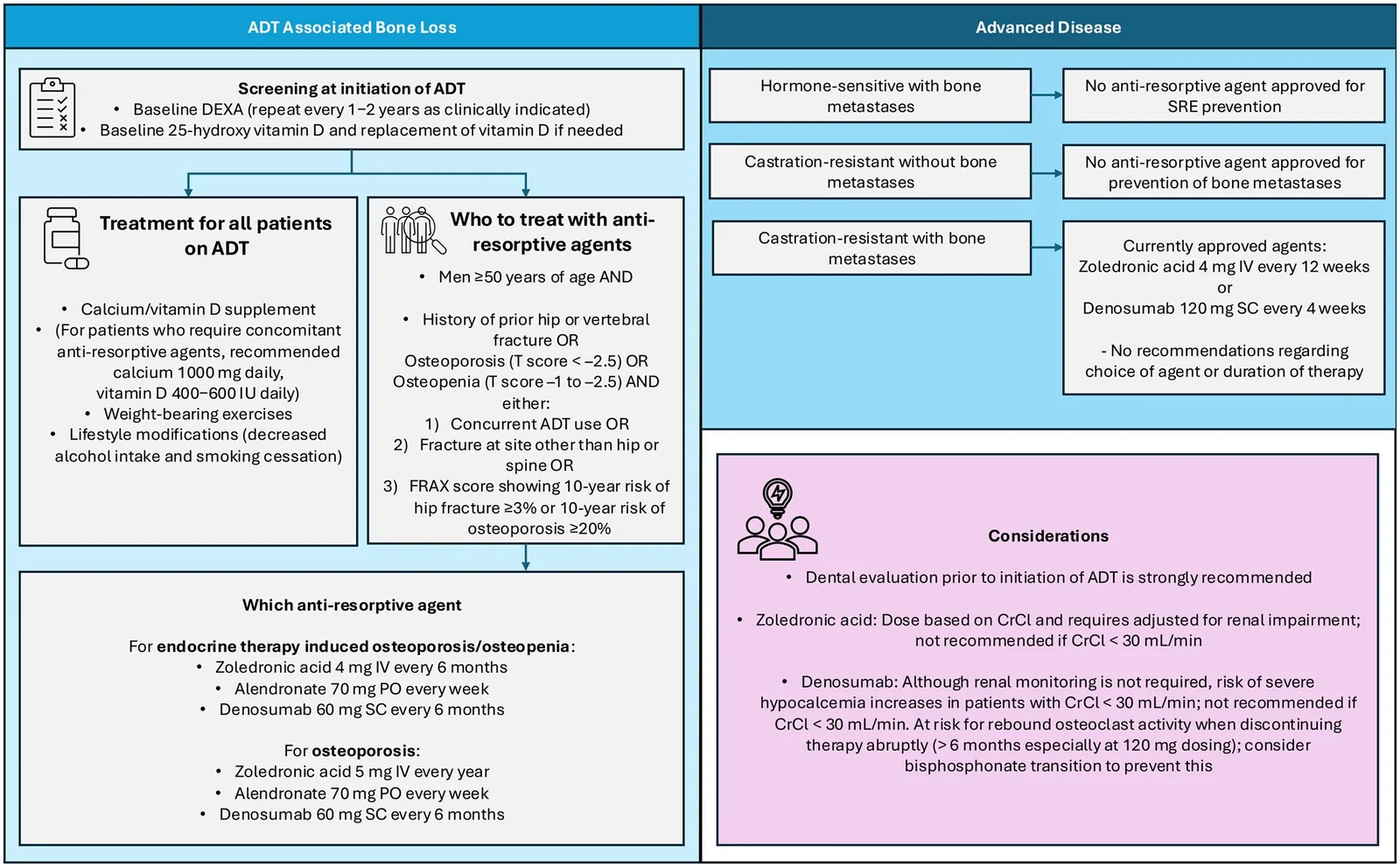
Management algorithm for bone health in prostate cancer. BHAs (zoledronic acid and denosumab) are not approved for use in mHSPC with bone metastases or CRPC without bone metastases. ADT, androgen-deprivation therapy; BHA, bone health agent; CrCl, creatine clearance; CRPC, castration-resistant prostate cancer; DEXA, dual-energy X-ray absorptiometry; FRAX, Fracture Risk Assessment Tool; IU, international units; IV, intravenous; mHSPC, metastatic hormone-sensitive prostate cancer; PO, orally; SC, subcutaneous; SRE, skeletal-related event.

Taking these considerations into account, we propose a test-and-treat algorithm for bone health management in men with prostate cancer (see Figure 2; Figure S1).

## Discussion

Bone health is a critical consideration in the management of men with prostate cancer. Both the cancer itself and its treatments can compromise bone integrity, increasing fracture, and SRE risk. Bone metastases are almost ubiquitous in men with mCRPC, and bone metastasis-related factors such as SSE occurrence, metastatic volume, and pain intensity may predict survival.^107–109^ In light of the major complications associated with bone metastases and their impact on patients’ quality of life and burden on healthcare systems, prevention and treatment of SSEs is critical. Some authors argue that bone metastases are an important driver of treatment decisions in patients with mCRPC.^110^ Prevention of SREs has only been evaluated in a few prostate cancer therapies. Improving treatment-related bone loss can take place across the prostate cancer continuum, even in localized disease, by supporting men to make appropriate and beneficial lifestyle changes and initiate BHAs alongside bone-targeted prostate cancer therapies where appropriate.

In mCRPC, BHAs are essential for all men with bone metastases for treatment-related bone loss and prevention of SREs.^17^ The role of BHAs in mHSPC is less established. Current recommendations support their use for managing treatment-related bone loss, but not for SRE prevention, as there is little evidence of clinical benefit in this context.^17^^,^^66^ A recent propensity-matched analysis identified 505 pairs of patients with mHSPC and bone metastases receiving versus not receiving denosumab or zoledronic acid.^111^ Deaths occurred in 163 (32.9%) in the bone-modifying agent (BMA) group vs 129 (26.0%) in the non-BMA group (*P* = .018); however, Kaplan–Meier time-to-event analysis was not significant (HR, 1.06; 95% CI, 0.84-1.34; *P* = .609). No significant effect was seen on SREs, including pathologic fracture or spinal cord compression. In a nested analysis in 169 pairs of patients, zoledronic acid and denosumab demonstrated similar survival and acute kidney injury risk. Nonetheless, BHAs may offer benefits in selected men with mHSPC at high risk of skeletal complications, guided by individualized assessment. Despite clear evidence and guidelines supporting BHA use,^17^ real-world studies consistently show underuse.^18–21^ The disparity in BHA prescribing patterns suggests a need for targeted education interventions and standardized protocols. Implementation strategies should focus on creating clear pathways for early BHA initiation, particularly within the critical window of starting life-prolonging therapies. The emergence of multiple effective prostate cancer therapies that reduce SREs (abiraterone, enzalutamide, radium-223) has created opportunities and challenges in optimizing treatment sequencing. Recent evidence from the PEACE-III study demonstrates the importance of concomitant BHA use in patients receiving treatment for mCRPC, particularly with enzalutamide or enzalutamide plus radium-223.^86^

To appropriately manage and mitigate BHA-associated risks, systematic monitoring protocols for adverse events should be implemented, particularly considering recent FDA warnings for patients with CKD. Clear guidelines outlining when and how BHAs should be de-escalated in patients with treated or non-active bone metastases are also needed. This is of particular importance for denosumab-treated patients who may be at risk of rebound fractures. Prospective studies examining the optimal duration of bone-targeted therapy may aid the creation of standardized protocols and treatment guidelines.

There are currently few biomarkers that accurately predict the impact of treatment on bone health and management of metastases.^112^ In the future, multi-omic techniques may help to define treatment targets for individuals across the course of their prostate cancer. Future treatment options may also help in the management of bone metastases, for example, by targeting cytotoxic treatment more precisely to bone metastases. While radiation therapy is primarily used to provide pain relief for patients with symptomatic bone metastases, there is increasing interest in prophylactic external beam radiotherapy (EBRT) for asymptomatic bone metastases.^113^^,^^114^ In a recent randomized, phase II trial, comparing EBRT and standard of care for asymptomatic bone metastases, EBRT was associated with a significant reduction in SREs at 1 year and significantly prolonged overall survival at 2.5 years.^114^

In conclusion, bone loss is an important source of morbidity and impaired quality of life in men with prostate cancer. Minimizing treatment-related bone loss across the prostate cancer continuum, including mHSPC, and preventing SSEs in patients with mCRPC should be standard practice to improve health outcomes and quality of life. This should include early risk assessment and multidisciplinary intervention with appropriate pharmacologic treatment and lifestyle changes. Targeted educational interventions, standardized guidelines, and improved tools to identify high-risk patients are necessary to aid clinical practice and bone health management decisions. The graphical abstract presents a simple summary of key points to bear in mind. Please also see the accompanying vlog in which Dr McKay discusses the key points of this review.

## Supplementary Material

oyag321_Supplementary_Data

## Data Availability

No new data were generated or analyzed in support of this narrative review of existing literature.
