# Supplementary material for: Bone health in advanced prostate cancer: pathophysiology and management strategies
Source: Oncologist. 2026 Aug 25;31(9):oyag321. doi: 10.1093/oncolo/oyag321 (PMC13548789; doi:10.1093/oncolo/oyag321)
Supplement: Supplementary file 1 [file oyag321_supplementary_data.zip › Supplementary Figure.pdf]

Bone health in advanced prostate cancer: Pathophysiology and management strategies

Risk factors for poor bone health in men with prostate cancer

- Bone metastases stimulate excess bone resorption
- ADT leads to reduced BMD and increased fracture risk
- Increased age
- Lifestyle (poor diet, smoking, alcohol intake, lack of exercise)

Who to screen for bone loss

All men starting or receiving ADT for prostate cancer, regardless of disease stage

Screening options for bone loss

- Fracture Risk Assessment Tool
- Baseline DEXA (repeat every 1–2 years as clinically indicated), alone, or with Trabecular Bone Score
- Dental evaluation
- Baseline 25-hydroxy vitamin D

Treatment algorithm for patients with ADT-associated bone loss

Screening at initiation of ADT

Treatment for all patients on ADT

- Calcium/vitamin D supplement
- For patients who require concomitant antiresorptive agents, recommend calcium 1000 mg daily, vitamin D 400–600 IU daily
- Weight-bearing exercises
- Lifestyle modifications (decreased alcohol intake and smoking cessation)

Who to treat with antiresorptive agents

- Men ≥ 50 years of age AND
- History of prior hip or vertebral fracture OR
  Osteoporosis (T score < −2.5) OR
  Osteopenia (T score −1 to −2.5) AND either:
  1. Concurrent ADT use OR
  2. Fracture at site other than hip or spine OR
  3. FRAX score showing 10-year risk of hip fracture ≥ 3% or 10-year risk of osteoporosis ≥ 20%

Which antiresorptive agent?

For **endocrine therapy-induced osteoporosis/osteopenia:**

- Zoledronic acid 4 mg IV every 6 months
- Alendronate 70 mg PO every week
- Denosumab 60 mg SC every 6 months

For **osteoporosis:**

- Zoledronic acid 5 mg IV every year
- Alendronate 70 mg PO every week
- Denosumab 60 mg SC every 6 months

Treatment algorithm for patients with advanced disease

Hormone-sensitive with bone metastases

- No antiresorptive agent approved for SRE prevention

Castration-resistant without bone metastases

- No antiresorptive agent approved for prevention of bone metastases

Castration-resistant with bone metastases

Currently approved agents:

Zoledronic acid 4 mg IV every 12 weeks OR

Denosumab 120 mg SC every 4 weeks

No recommendations regarding choice of agent or duration of therapy. Stoppage of denosumab should be performed with caution since this will result in loss of effect within 6 months resulting in rapid bone loss and a possible increase in fracture risk

Considerations

- Zoledronic acid: Dose based on CrCl and requires adjustment for renal impairment; not recommended if CrCl < 30 mL/min
- Denosumab: Although renal monitoring is not required, risk of severe hypocalcemia increases in patients with CrCl < 30 mL/min; not recommended if CrCl < 30 mL/min. At risk for rebound osteoclast activity when discontinuing therapy abruptly (> 6 months especially at 120 mg dosing); consider bisphosphonate transition to prevent this

Ongoing care

Manage and mitigate bone health agent-associated risks with the wider multidisciplinary team, including frequent dental evaluations

De-escalate bone health agent dosing frequency if patients are no longer at risk of fracture

ADT, androgen-deprivation therapy; BMD, bone mineral density; CrCl, creatinine clearance; DEXA, dual-energy X-ray absorptiometry; FRAX, Fracture Risk Assessment Tool; IU, international unit; IV, intravenous; PO, orally; SC, subcutaneous; SRE, skeletal-related event
